# A Case of a Gastric Stent Complication Presenting With a Gastrojujenal Fistula

**DOI:** 10.7759/cureus.51143

**Published:** 2023-12-26

**Authors:** Sarah A Breakeit, Sultanah Gheshayan, Bader A Alamri, Emad F Albalwi, Nasser A Alharbi, Sultan Alhabdan

**Affiliations:** 1 General Surgery, King Abdulaziz Medical City, Riyadh, SAU; 2 College of Medicine, King Saud bin Abdulaziz University for Health Sciences, Riyadh, SAU; 3 General Surgery, College of Medicine, King Saud bin Abdulaziz University for Health Sciences, Riyadh, SAU; 4 General and Colorectal Surgery, College of Medicine, King Saud bin Abdulaziz University for Health Sciences, Riyadh, SAU

**Keywords:** complications, leakage, gastrojujenal fistula, sleeve gastrectomy, bariatric surgery

## Abstract

Laparoscopic sleeve gastrectomy (SG) is a widely performed bariatric procedure known for its safety and efficacy, yet complications, particularly postoperative leaks, remain a concern. Endoscopic stenting is one of the approaches for leak management that has some complications such as the rare fistula with adjacent organs. Here, we present a unique case of a 56-year-old diabetic female who developed a gastroenteric fistula following endoscopic stent placement for a post-SG leak. The patient had a delayed stent removal, and three weeks later she presented with dysphagia and vomiting in a follow-up appointment. Esophagogastroduodenoscopy (EGD) showed an esophageal stricture and a large gastroenteric fistula that was confirmed by imaging to be a fistula between the pylorus and proximal small bowel. The patient's symptoms improved gradually with conservative management, and imaging showed the resolution of the fistula and associated esophageal stricture. This case highlights the importance of timely stent removal (ideally within 6-8 weeks) and close follow-up with EGD and CT to detect and manage potential complications, while emphasizing the need for further research on optimal stent placement duration.

## Introduction

Laparoscopic sleeve gastrectomy (SG) has emerged as the most-performed bariatric procedure according to the IFSO (International Federation for the Surgery of Obesity and Metabolic Disorders) [1]. While it is generally considered a safe procedure, complications may occur, such as severe reflux, pneumonia, vomiting, and leak, with post-SG leakage being the most concerning and morbid complication [2]. 

There are multiple treatment methods for leaks described in the literature, such as endoscopic clipping, usage of fibrin glue, and endoscopic stenting, with stenting being the most common approach due to its efficacy and advantages like providing an opportunity for wound healing with continued oral intake. Complications associated with stents are uncommon, yet can be morbid, such as esophageal stenosis, upper gastrointestinal bleeding, and rarely fistulas with adjacent organs. 

In this paper, we report a rare complication of endoscopic stenting for a post-SG leak that resulted in a gastroenteric fistula.

## Case presentation

**Figure 1:**
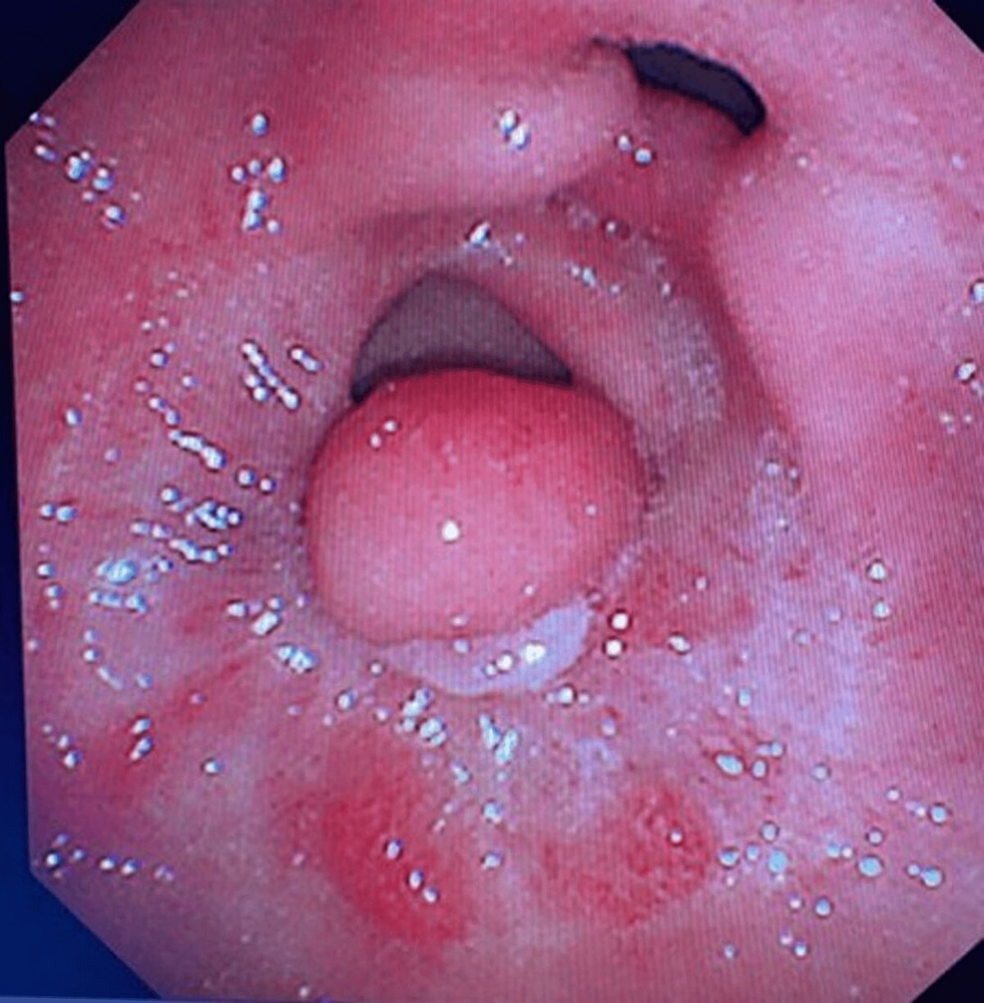
EGD showing a gastroenteric fistula. EGD: Esophagogastroduodenoscopy

A 56-year-old diabetic female presented to the emergency department with severe nonradiating epigastric pain for one day. She is post-SG on day 7. CT showed a 6.7 cm perigastric collection with a contrast leak within the collection. The patient underwent uneventful endoscopic stent placement using a 28 mm x 230 mm gastroesophageal covered stent. She was discharged safely with a stent removal appointment after six weeks; however, she had a delayed appointment, and a stent was removed under fluoroscopic guidance approximately eight weeks after (52) days with no contrast leak. The patient was seen three weeks later in the outpatient clinic for a follow-up. She complained of dysphagia to solid food and frequent vomiting, for which she was scheduled for an esophagogastroduodenoscopy (EGD). EGD (Figure 1) showed an esophageal stricture that was managed by balloon dilatation and a large gastroenteric fistula just around the pylorus with hypertrophic nodules proximal to it.

**Figure 2:**
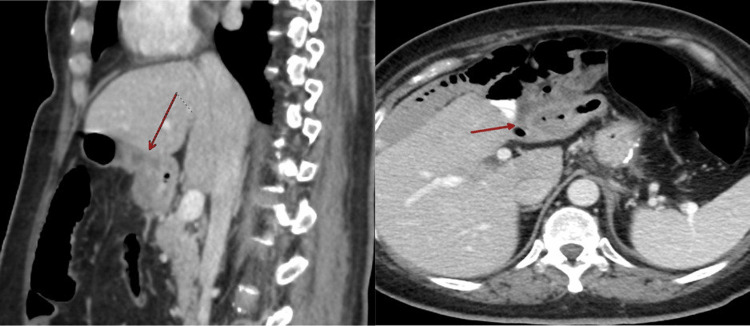
CT abdomen showing the gastrojejunal fistula (red arrow).

The fistula tract was identified with abdominal CT (Figure 2) just around the pylorus and proximal small bowel. The patient’s symptoms improved gradually with conservative management, and repeated scans showed resolution of the fistula. The esophageal stricture as well has resolved after multiple EGD dilatations. The patient was seen in the clinic weeks after the last EGD dilatation and CT scan. The patient was tolerating oral intake well with no more dysphagia or vomiting, and she achieved optimal excess weight loss.

## Discussion

Laparoscopic SG currently is the most performed bariatric procedure for obesity based on the IFSO [1]. SG has grown in popularity due to its safety, relative technical ease, and efficacy in achieving optimal excess weight loss and remission of associated metabolic comorbidities. Nevertheless, the safety of sleeve gastrectomy has been greatly reported, yet complications such as bleeding, thromboembolic events, and leaks might occur [2,3]. Leak at the angle of His is the most concerning adverse event due to the linked morbidity and mortality. Various pathophysiological explanations for the leak have been described in the literature, one of which is variation in pressure due to uneven staple line, tight incisura, or twist [4-6]. The treatment for post-SG leakage may include surgical or endoscopic drainage; endoscopic management including clipping and stenting; and rarely surgical intervention and revisional bariatric surgery [7]. Endoscopic stenting is the most common approach used for leak management due to its feasibility and efficacy, especially when there is a morphological abnormality of the sleeved stomach requiring stenting to even the intraluminal pressure. Moreover, the preference for using stents in leak management is that they provide an opportunity for wound healing by bypassing the leak site while allowing oral intake and avoiding surgical intervention if possible. Studies reported that the most adequate time for stent removal is between 6 and 8 weeks, and the success rate associated with stenting is 80%-94% [8-10].

Despite the abovementioned advantages, side effects and complications can occur commonly: nausea, epigastric pain, satiety, hyperhidrosis, and the tendency of stents toward distal migration [11-13]. Less common yet morbid complications have been reported such as massive bleeding, perforation, and fistula. In this paper, we reported a rare complication in post-SG patients as there was a formation of a gastrojejunal fistula noted during the endoscopic removal of the stent that was delayed from the desired date. The duration for stent removal in our center is 4 to 6 weeks; yet due to logistic reasons the removal for this patient had to be delayed till almost eight weeks. The hypothesized pathophysiology in this case mostly linked to the prolonged pressure effect from the stent over adjacent intestines. The type and size of the stent could be added causes to the duration, as the exact type has been used with other patients in the initiation yet shorter duration. A gastroenteric fistula due to other etiology has been described in the literature such as bowel obstruction, inflammatory bowel disease, radiation, and ingestion of corrosive agents [14]. Prior studies poorly reported such an event which could be due to its rarity. The management of a gastroenteric fistula depends on the etiology and associated symptoms. In our case, symptoms were the indicator of an intervention as the stent was removed which was the causing factor. As symptoms in our patient were mild and tolerable, no other intervention was offered other than observation. Symptoms and imagiological findings of the fistula were solved during follow-up.

As there was no prior occurrence in our institution, this incident has brought our attention to the importance of the stent placement duration. Further consideration for a shorter duration is currently encouraged, yet larger studies are needed to identify the optimal stent placement duration.

## Conclusions

Although laparoscopic SG is the most performed bariatric procedure for obesity and is recognized for its safety and efficacy, it can present with uncommon complications like our case. We presented a case of a gastroenteric fistula which is a very rare condition postendoscopic stenting for SG leak. A shorter duration for stent placement is advisable, and re-evaluation for postremoval healing and complications with radiological or endoscopic follow-ups is crucial. This case emphasizes the need for more awareness and research to figure out the best time to remove stents and further studies to improve how we handle complications after laparoscopic SG.

## References

[1] Angrisani L, Santonicola A, Iovino P, Ramos A, Shikora S, Kow L (2021). Bariatric surgery survey 2018: similarities and disparities among the 5 IFSO chapters. Obes Surg.

[2] Chang SH, Freeman NL, Lee JA, Stoll CR, Calhoun AJ, Eagon JC, Colditz GA (2018). Early major complications after bariatric surgery in the USA, 2003-2014: a systematic review and meta-analysis. Obes Rev.

[3] Dang JT, Shelton J, Mocanu V, Sun W, Birch DW, Karmali S, Switzer NJ (2021). Trends and outcomes of laparoscopic sleeve gastrectomy between 2015 and 2018 in the USA and Canada. Obes Surg.

[4] Guzman-Pruneda FA, Brethauer SA (2021). Gastroesophageal reflux after sleeve gastrectomy. J Gastrointest Surg.

[5] Vilallonga R, Hidalgo M, Garcia Ruiz de Gordejuela A (2020). Operative and postoperative complications of laparoscopic sleeve gastrectomy in super and nonsuper obese patients: a center of excellence experience comparative study. J Laparoendosc Adv Surg Tech A.

[6] Schulman AR, Thompson CC (2017). Complications of bariatric surgery: what you can expect to see in your GI practice. Am J Gastroenterol.

[7] de Aretxabala X, Leon J, Wiedmaier G (2011). Gastric leak after sleeve gastrectomy: analysis of its management. Obes Surg.

[8] Eisendrath P, Cremer M, Himpens J, Cadière GB, Le Moine O, Devière J (2007). Endotherapy including temporary stenting of fistulas of the upper gastrointestinal tract after laparoscopic bariatric surgery. Endoscopy.

[9] Walsh C, Karmali S (2015). Endoscopic management of bariatric complications: a review and update. World J Gastrointest Endosc.

[10] Fukumoto R, Orlina J, McGinty J, Teixeira J (2007). Use of Polyflex stents in treatment of acute esophageal and gastric leaks after bariatric surgery. Surg Obes Relat Dis.

[11] Salinas A, Baptista A, Santiago E, Antor M, Salinas H (2006). Self-expandable metal stents to treat gastric leaks. Surg Obes Relat Dis.

[12] Edwards CA, Bui TP, Astudillo JA (2008). Management of anastomotic leaks after Roux-en-Y bypass using self-expanding polyester stents. Surg Obes Relat Dis.

[13] Eubanks S, Edwards CA, Fearing NM (2008). Use of endoscopic stents to treat anastomotic complications after bariatric surgery. J Am Coll Surg.

[14] Farooqi N, Tuma F (2023). Intestinal fistula. StatPearls [Internet].

